# Activity spaces in studies of the environment and physical activity: A review and synthesis of implications for causality

**DOI:** 10.1016/j.healthplace.2019.04.003

**Published:** 2019-07

**Authors:** Lindsey Smith, Louise Foley, Jenna Panter

**Affiliations:** a MRC Epidemiology Unit & UKCRC Centre for Diet and Activity Research (CEDAR), University of Cambridge, School of Clinical Medicine, Box 285, Cambridge Biomedical Campus, Cambridge, Cambridgeshire, CB2 0QQ, UK; b National Institute for Health Research (NIHR) Global Health Research Group and Network on Diet and Activity, University of Cambridge, School of Clinical Medicine, Box 285, Cambridge Biomedical Campus, Cambridge, Cambridgeshire, CB2 0QQ, UK

**Keywords:** Activity space, Environment, Physical activity, Individual exposure, Research methods, Causal inference

## Abstract

Activity spaces are increasingly used to understand how people interact with their environment and engage in activity but their use may raise challenges regarding causal inference. We conducted a systematic review of findings and the methodological, analytical and conceptual issues relevant to causal inference. Studies were included if they comprised a spatial summary of locations visited, assessed any part of the causal pathway between the environment, physical activity and health, and used quantitative or qualitative methods. We searched seven electronic databases in January 2018 and screened 11910 articles for eligibility. Forty-seven studies were included for review. Studies answered research questions about features of or environmental features within activity spaces using a range of spatial and temporal summary techniques. The conceptual challenge of using activity spaces to strengthen causal inference was rarely considered, although some studies discussed circularity, temporality, and plausibility. Future studies should use longitudinal and experimental designs and consider the potential and actual use of spaces for physical activity, and their relationship with total levels of activity.

## Highlights

• We reviewed studies that investigated the environment-physical activity relationship using activity spaces.
• Studies assessed the features of (shape or size) or within activity spaces.
• A range of spatial and temporal methods were employed to define activity spaces and answer different research questions.
• Most studies were cross-sectional and casual inference was rarely discussed.
• Evaluation of interventions using longitudinal or qualitative data would be useful to strengthen causal inference.

## 1 Introduction

Physical activity reduces the risk of chronic disease (Haskell et al., 2007; World Health Organization, 2010) and a substantial proportion of the population would benefit from being more active (Hallal et al., 2012). Public health strategies increasingly identify the environment as a modifiable determinant of activity. For example, the World Health Organisation Global Action Plan on physical activity identifies the importance of improvements to walking and cycle networks, road safety, and access to public open spaces and the need to understand where people live, work and play for their effective delivery (World Health Organization, 2018). Previous studies that investigated the relationship between characteristics of the environment and activity have predominantly examined the residential neighbourhood, applying static administrative boundaries or buffers around participants' addresses (Chaix, 2009; McCormack and Shiell, 2011; Van Cauwenberg et al., 2011). These assessments do not characterise the spaces within which people actually move and are exposed to, or account for within- and between-person heterogeneity in spatial habits (Perchoux et al., 2013). Furthermore, assessing the environment around the home address can create spatial and temporal uncertainty relating to actual exposure (the uncertain geographic context problem (UGCP)) because it is unknown how much time people spend in those environments (Kwan, 2012).

One concept which aims to more accurately capture exposure to different environments is the activity space. The general principle of an activity space is that it provides a dynamic measure of the environment by describing the locations and spaces an individual interacts with as a result of their activities (Golledge and Stimson, 1997). It is organised around key anchor points including home and work locations and extends to locations such as food outlets, child's schools, parks, and social meeting points (Axhausen et al., 2002). Locations may be weighted by the frequency, regularity, and duration at which they are visited (Perchoux et al., 2013). The concept of the activity space was introduced in 1970 when space-time geography was used to assess daily travel behaviours (Hägerstraand, 1970) and has since been applied in a number of disciplines including transport, psychology, and food environments, using methods including diaries, GPS devices, web mapping applications and interviews (Schönfelder and Axhausen, 2003; Harding et al., 2012). With an increasing shift towards objective assessment of activity and behaviours, the number of studies using GPS devices to examine the associations between environments and activity has grown in recent years (Krenn et al., 2011; Chaix et al., 2013; James et al., 2016).

Chaix and colleagues recognise that studies applying the concept of the activity space have the potential to strengthen the basis for causal inference between the environment and physical activity, if the methods and research question are thoughtfully implemented (Chaix et al., 2013). Some methods used to derive activity spaces capture environments potentially accessible to an individual over time but also capture environments regardless of a person's use of, awareness or exposure to that environment. Others methods describe places visited or spaces used for physical activity. However, an individual's preference to perform an activity may bias any associations observed between accessibility to these environments and physical activity because people who want to be active seek out environments or locations supportive of activity in order to be active. Using spaces used for activity as a measure of accessible environments gives rise to a circular argument as an individual would not have visited the location if they did not intend to be active there. This circularity may lead to a form of confounding called selective daily mobility bias which is likely to generate problems for causal inference because certain individuals may appear more exposed and any relationship between accessibility to these spaces and physical activity behaviours may be strengthened.

A previous review described the origins of the concept of an activity space, the categorisation of the disciplinary research areas, and the methods used (Patterson and Farber, 2015). This previous narrative review is not systematic and does not clearly outline the approach used to search for articles. It is also mainly descriptive and does not develop or evaluate the concepts in depth. Consequently, there is a gap in understanding how the activity spaces have been applied and used in existing research. This review aims to examine the application of the activity space in studies of the environment and physical activity, identify what methods have been used, the research questions addressed, synthesise the methodological, analytical and conceptual issues, and assess the extent to which they strengthen the basis for causal inference.

## 2 Methods

### 2.1 Search strategy

To inform the design and scope of our search strategy and inclusion criteria, we completed pilot searches. We identified existing systematic reviews relating to GPS-located physical activity (Maddison and Ni Mhurchu, 2009; Krenn et al., 2011; Mccrorie et al., 2014; Loveday et al., 2015; Cetateanu and Jones, 2016) and extracted key words and terms of interest from each. We then tested terms relating to themes common to the reviews (GPS, environment, activity space and physical activity) in PubMed. Different combinations of the themes were tested to understand if any search terms limited the results. Titles of studies from the searches were screened and potentially relevant articles were shortlisted and reviewed by all authors to inform the final search strategy and inclusion criteria. Further details of the minor changes made as a result of pilot searches are described in supplementary material (Appendix 1).

In January 2018, we completed systematic searches of seven electronic databases to identify potential literature searching for articles published before 20th January 2018 (PubMed, Web of Science, TRID (Transport Research International Documentation), Scopus, Ovid MEDLINE, ProQuest, NICE (National Institute for Health and Care Excellence) evidence search). Search terms were based on four themes (1) mapping; (2) activity space; (3) physical activity; and (4) health/behaviour (Table 1) and the full search strategy as implemented in PubMed is detailed in the supporting material (Appendix 1). In each section we included both broad (e.g. physical activity; exercise) and more specific search terms (e.g. walking; cycling) to ensure good coverage. Given the volume of outputs from each database, restrictions were applied to limit the results to studies of human behaviour in the multidisciplinary databases.

**Table 1:** Search terms.

Theme	Search terms
(1) Mapping	GPS, GIS, map, behavioural geography, context
(2) Activity space	Activity space, potential path, daily path, destinations
(3) Physical Activity	Physical activity, walking, cycling, exercise, transport, mobility, movement
(4) Health/behaviour	Spatial behaviour, health behaviour, community, social cohesion
Search query:	1 AND 2 AND (3 OR 4)

To identify additional relevant literature, eligible articles were forwards and backwards referenced searched by reviewing reference lists and papers that cited included studies. We also contacted the first and last authors of eligible articles with multiple publications (n = 10) via email and asked if they were aware of any other eligible articles. We also searched past editions of the GPS-Health Research Network (GPS-HRN) newsletter and emailed the editor to identify other relevant studies.

We registered our protocol with the International Prospective Register for Systematic Reviews (PROSPERO) in January 2018 (record number: CRD42018087095) (Smith et al., 2018).

### 2.2 Inclusion criteria

As we sought to understand how activity spaces had been used in studies assessing the association between the environment and activity, we took an inclusive approach embracing evidence on one potential causal pathway, drawing on the principles of a perspective articulated by Zenk (Zenk et al., 2011). This pathway might work in the following way: environmental characteristics in areas where people spend time might be associated with use of those environments (often captured through activity spaces) which might be related to levels of physical activity and subsequent health. Therefore, studies assessing environments exposed to as a result of use of space, characteristics of that space, physical activity, activity behaviours, or health outcomes were included.

We included all types of study designs, but studies had to use a spatial summary measure of movement, behaviour, activity, or locations visited and explicitly geo-locate spaces visited. The unit of analysis had to be the individual level and unique spatial summaries (activity spaces) must have been derived for each study participant. Locations could be self-reported and subsequently mapped or directly inferred from objective measures, such as GPS devices. Sub-sets of behaviour such as walking or trips made for a specific purpose were also included. No date, location, age, sample size, language or quality restrictions were applied.

Chaix and colleagues previously identified studies which assess the distribution of activity in different types of spaces or land use types, such as the time spent active in schools or parks, as descriptive and potentially limiting (Chaix et al., 2013). To focus the review, we excluded these descriptive studies. We also excluded studies that modelled or estimated routes, such as those that assumed individuals took the most direct route between two destinations, or described possible methods and did not apply them in an empirical study.

### 2.3 Study screening and data extraction

The lead author (LS) screened titles and abstracts for eligibility. In phase 1 of screening, all articles with obviously irrelevant titles and abstracts were excluded. In phase 2 of screening, consideration was given to the definition and concept of the activity space by all authors. All articles considered to provide potential context or methods of interest were initially grouped into one of six categories (Table 2). Articles in categories 1 to 5 were excluded and all articles in category 6 were retrieved in full text and reviewed by LS.

**Table 2:** Study categories identified from phase 2 of screening.

Category	Description
1	Studies where there was no variation in access for participants: • Those that assessed activity within a constrained area, such as internal environments (shopping centre, care home) or housing developments • Those that used non-continuous and infrequent locational data such as the mapping of social media check-ins • Those that described activity by environmental feature or land use type with no spatial summary
2	Studies that assessed non-physical activity outcomes where the causal pathway between environment and physical activity was unclear
3	Studies that assessed populations with long term limiting health conditions such as those with mobility disabilities or visual impairments
4	Non-empirical studies such as systematic reviews
5	Studies that modelled transport, such as those that map taxi or freight journeys, following the vehicle's route rather than the individual's
6	Empirical or methodological studies relating to activity spaces, environments, and physical activity

Language translation programmes were used and expertise from colleagues was sought to translate articles not written in English. 20% of articles identified for full text review were screened independently by LF for agreement. Reasons for exclusion were recorded by both authors and any discrepancies in agreement were referred to JP for a majority decision.

Information on study design, sample characteristics, research questions, activity space delineations, exposure and outcome measures, key findings and conceptual discussions related to causality were extracted from eligible articles by LS into pre-designed forms (Appendix 1, Table A1). In doing so, the terms and delineations used by the original authors were extracted. LF checked data extractions for accuracy and completeness for 20% of articles.

### 2.4 Data synthesis

We categorised studies according to the spatial and temporal methods used to define and delineate activity spaces, research questions addressed, and how activity spaces were applied to investigate which parts of the potential causal pathway between environmental exposure and physical activity. These categories were informed by data extracted from the studies and were designed to provide insight on the ways the activity space had been applied practically and conceptually. We synthesised results narratively to understand the consideration given to causal inference framed by Bradford Hill's principles of causation (Hill, 1965) and to identify any gaps in the field. Although other statistical frameworks for causality were considered (Pearl, 2009; Imbens and Rubin, 2016), the principles from Bradford Hill were chosen due to their broad nature and relevance to epidemiological studies.

## 3 Results and discussion

### 3.1 Study selection

**Fig. 1:**
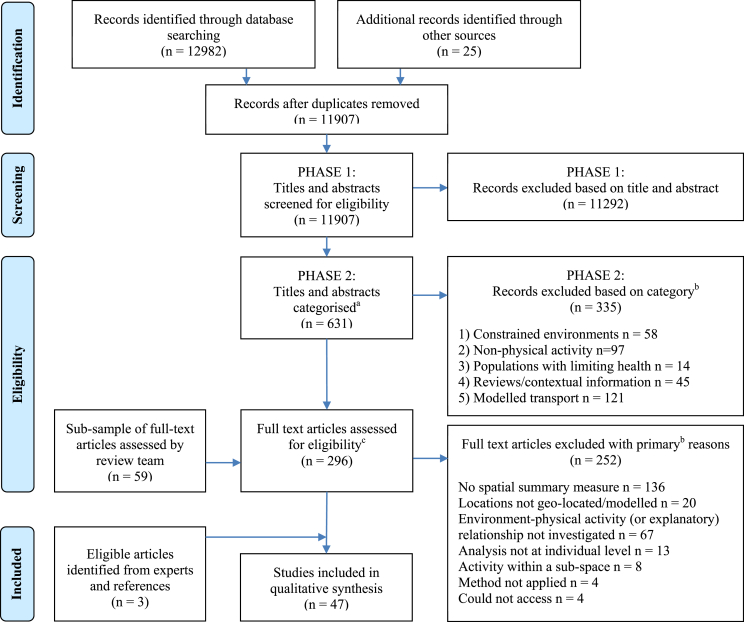
*Study selection*. ^a^See Table 2 for details on categories ^b^Some studies met multiple criteria for exclusion. Categories and reasons for exclusion were ordered and only the criterion of highest order is shown ^c^All articles from category six (see Table 2).

The electronic database searches returned 12982 records and 25 records were identified from GPS-HRN newsletters. After screening titles and abstracts and categorizing articles of potential interest, 296 were identified for full text review. LF reviewed 20% of the full text articles with a 92% agreement rate. Five articles were referred to JP; there were no patterns in the reasons for referral. Three articles were identified from forwards and backwards reference searches. 8/10 of experts responded and did not provide any additional eligible articles. In total, 47 articles met the inclusion criteria. The process of article inclusion and are detailed in Fig. 1 and full a list of included articles and detailed tables are provided in Appendix 2.

### 3.2 Study characteristics

All articles were published after 2007 with 25 published within the past three years (2016–2018). The majority of study populations originated from high income countries, primarily from cities or metropolitan neighbourhoods in North America (n = 24) and Europe (n = 17). One study was identified from a middle income country, drawing on a sample from 28 villages near one city in India (Sanchez et al., 2017), and one studied rural dwellers from three towns in Northern Ireland (Kamruzzaman and Hine, 2012). Samples were studied for all age groups with most drawn from adult (n = 22) populations. Some targeted females (Rodríguez et al., 2012; Rodriguez et al., 2015), lower income participants (Hirsch et al., 2016; Franke et al., 2017), university members (Kamruzzaman et al., 2011; Vich et al., 2017), e-bike owners (Plazier et al., 2017), or those living in subsidised housing (Colabianchi et al., 2014). Most studies were solely cross-sectional in design (n = 43) and four assessed activity spaces in relation to an intervention (Kamruzzaman et al., 2011; Kosaka et al., 2014; Chaix et al., 2017; Tribby et al., 2017). All intervention studies examined alterations to the built environment including access to a demand responsive transport service, improvements to street safety, a covered walkway, and a modelled increase in services in the residential area. Study characteristics are detailed in Table 3.

**Table 3:** Summary of study characteristics.

Characteristic	Reference	No.
**Continent**		
N America	(Zenk et al., 2011) (Rodriguez et al., 2015) (Rodríguez et al., 2012) (Hirsch et al., 2016) (Hirsch et al., 2014) (Franke et al., 2017) (Colabianchi et al., 2014) (Tribby et al., 2017) (Rundle et al., 2016) (Burgoine et al., 2015) (Chen et al., 2017) (Lee et al., 2016) (Fan, 2007) (Loebach and Gilliland, 2016a) (Quistberg et al., 2017) (Houston, 2014) (Larsen et al., 2009) (Hand et al., 2018) (Larsen et al., 2016) (Loebach and Gilliland, 2016b) (van Heeswijck et al., 2015) (Kang et al., 2017) (Harding, 2013) (Chen and Akar, 2016)	24
Europe	(Kamruzzaman and Hine, 2012) (Vich et al., 2017) (Kamruzzaman et al., 2011) (Plazier et al., 2017) (Chaix et al., 2017) (McMinn et al., 2014) (Helbich, 2017) (Helbich et al., 2016) (Duncan et al., 2016) (Chaix et al., 2016) (Bell et al., 2015a) (Meijering and Weitkamp, 2016) (Milton et al., 2015) (Bell et al., 2015b) (Perchoux et al., 2014) (Hasanzadeh et al., 2017) (Wheeler et al., 2010)	17
Australia	(Villanueva et al., 2012) (Babb et al., 2017) (Vine et al., 2012)	3
Asia	(Sanchez et al., 2017) (Kosaka et al., 2014) (Yoo and Kim, 2017)	3
**Age group**		
Children	(Burgoine et al., 2015) (Loebach and Gilliland, 2016a) (Larsen et al., 2009) (Larsen et al., 2016) (Loebach and Gilliland, 2016b) (McMinn et al., 2014) (Helbich, 2017) (Helbich et al., 2016) (Wheeler et al., 2010) (Villanueva et al., 2012) (Babb et al., 2017)	11
Adolescents	(Rodriguez et al., 2015) (Rodríguez et al., 2012) (Colabianchi et al., 2014) (Lee et al., 2016)	4
Adults	(Sanchez et al., 2017) (Kamruzzaman and Hine, 2012) (Vich et al., 2017) (Kamruzzaman et al., 2011) (Plazier et al., 2017) (Chaix et al., 2017) (Kosaka et al., 2014) (Tribby et al., 2017) (Rundle et al., 2016) (Chen et al., 2017) (Quistberg et al., 2017) (Houston, 2014) (van Heeswijck et al., 2015) (Kang et al., 2017) (Harding, 2013) (Chen and Akar, 2016) (Duncan et al., 2016) (Chaix et al., 2016) (Bell et al., 2015a) (Bell et al., 2015b) (Perchoux et al., 2014) (Hasanzadeh et al., 2017)	22
Older adults	(Hirsch et al., 2016) (Hirsch et al., 2014) (Franke et al., 2017) (Hand et al., 2018) (Meijering and Weitkamp, 2016) (Milton et al., 2015) (Vine et al., 2012) (Yoo and Kim, 2017)	8
All	(Zenk et al., 2011) (Fan, 2007)	2
**Sample size**		
0–50	(Sanchez et al., 2017) (Franke et al., 2017) (Plazier et al., 2017) (Lee et al., 2016) (Hand et al., 2018) (Loebach and Gilliland, 2016b) (McMinn et al., 2014) (Bell et al., 2015a) (Meijering and Weitkamp, 2016) (Milton et al., 2015) (Bell et al., 2015b) (Babb et al., 2017) (Vine et al., 2012) (Yoo and Kim, 2017)	14
51–100	(Zenk et al., 2011) (Hirsch et al., 2016) (Hirsch et al., 2014) (Kosaka et al., 2014) (Burgoine et al., 2015) (Houston, 2014) (Helbich, 2017) (Helbich et al., 2016)	8
101–500	(Kamruzzaman and Hine, 2012) (Rodriguez et al., 2015) (Rodríguez et al., 2012) (Vich et al., 2017) (Kamruzzaman et al., 2011) (Colabianchi et al., 2014) (Chaix et al., 2017) (Tribby et al., 2017) (Loebach and Gilliland, 2016a) (Duncan et al., 2016) (Chaix et al., 2016)	11
501–1000	(Rundle et al., 2016) (Quistberg et al., 2017) (Larsen et al., 2009) (Larsen et al., 2016) (Kang et al., 2017)	5
1001–5000	(Fan, 2007) (Perchoux et al., 2014) (Hasanzadeh et al., 2017) (Wheeler et al., 2010) (Villanueva et al., 2012)	5
>5000	(Chen et al., 2017) (van Heeswijck et al., 2015) (Harding, 2013) (Chen and Akar, 2016)	4
**Study design**^a^		
Cross-sectional	(Zenk et al., 2011) (Kamruzzaman and Hine, 2012) (Rodriguez et al., 2015) (Hirsch et al., 2016) (Hirsch et al., 2014) (Vich et al., 2017) (Kamruzzaman et al., 2011) (Plazier et al., 2017) (Colabianchi et al., 2014) (Chaix et al., 2017) (Kosaka et al., 2014) (Rundle et al., 2016) (Burgoine et al., 2015) (Chen et al., 2017) (Lee et al., 2016) (Fan, 2007) (Loebach and Gilliland, 2016a) (Quistberg et al., 2017) (Houston, 2014) (Larsen et al., 2009) (Hand et al., 2018) (Larsen et al., 2016) (Loebach and Gilliland, 2016b) (van Heeswijck et al., 2015) (Kang et al., 2017) (Harding, 2013) (Chen and Akar, 2016) (McMinn et al., 2014) (Helbich, 2017) (Helbich et al., 2016) (Duncan et al., 2016) (Chaix et al., 2016) (Bell et al., 2015a) (Meijering and Weitkamp, 2016) (Milton et al., 2015) (Bell et al., 2015b) (Perchoux et al., 2014) (Hasanzadeh et al., 2017) (Wheeler et al., 2010) (Villanueva et al., 2012) (Babb et al., 2017) (Vine et al., 2012) (Yoo and Kim, 2017)	43
Longitudinal	(Sanchez et al., 2017) (Franke et al., 2017) (Tribby et al., 2017)	3
Both	Rodríguez et al. (2012)	1
Intervention	(Kamruzzaman et al., 2011) (Chaix et al., 2017) (Kosaka et al., 2014) (Tribby et al., 2017)	4
**Analysis**		
Qualitative	(Plazier et al., 2017) (Hand et al., 2018) (Bell et al., 2015a) (Meijering and Weitkamp, 2016) (Milton et al., 2015) (Bell et al., 2015b) (Yoo and Kim, 2017)	7
Quantitative	(Zenk et al., 2011) (Sanchez et al., 2017) (Rodriguez et al., 2015) (Rodríguez et al., 2012) (Hirsch et al., 2016) (Hirsch et al., 2014) (Vich et al., 2017) (Kamruzzaman et al., 2011) (Colabianchi et al., 2014) (Chaix et al., 2017) (Kosaka et al., 2014) (Tribby et al., 2017) (Rundle et al., 2016) (Burgoine et al., 2015) (Chen et al., 2017) (Lee et al., 2016) (Fan, 2007) (Loebach and Gilliland, 2016a) (Quistberg et al., 2017) (Houston, 2014) (Larsen et al., 2009) (Larsen et al., 2016) (van Heeswijck et al., 2015) (Kang et al., 2017) (Harding, 2013) (Chen and Akar, 2016) (McMinn et al., 2014) (Helbich, 2017) (Helbich et al., 2016) (Duncan et al., 2016) (Chaix et al., 2016) (Perchoux et al., 2014) (Hasanzadeh et al., 2017) (Wheeler et al., 2010) (Villanueva et al., 2012)	35
Both	(Kamruzzaman and Hine, 2012) (Franke et al., 2017) (Loebach and Gilliland, 2016b) (Babb et al., 2017) (Vine et al., 2012)	5

a Column totals to more than 47 as some studies listed in more than category and categories not mutually exclusive.

## 4 Methods employed

### 4.1 Spatial extent of activity space

**Fig. 2:**
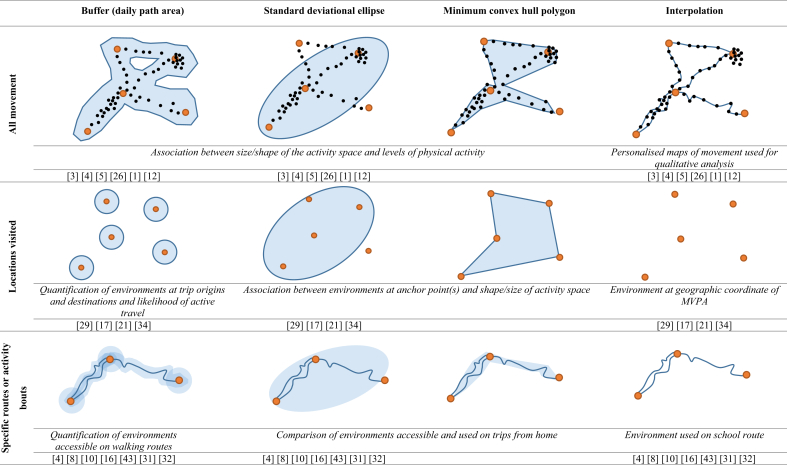
Methods used to delineate activity spaces with descriptions of example applications.  Anchor point (for example: home/work/school/sports club location). •/Geo-located movement.  Activity space.

Activity spaces were derived from objective GPS data (n = 30) and reported locational data (n = 24). Seven studies used both. Regardless of the method used, all studies assessed the spatial extent of activity in one of three ways: (i) by using all movement, (ii) by focusing on key locations visited or (iii) by focusing on specific routes or activity types (Fig. 2). There was a range of different methods employed within each broad grouping and sometimes within a single study.

#### 4.1.1 All movement (n = 20 studies)

Methods included daily path areas (DPA) (buffer of all points or tracks) (n = 6), standard deviational ellipses (SDE) (n = 5), minimum convex hull polygons (MCP) (n = 4), and personalised maps of plotted points or tracks of movements (n = 11). Some examples of the latter indicated areas accessed using different travel modes (Vine et al., 2012; Plazier et al., 2017) or for physical activity (Yoo and Kim, 2017). Unique estimations of the activity space also used a maximum path distance to recorded points of movement (Loebach and Gilliland, 2016a) and a composite measure of distances travelled and frequency at locations (Kamruzzaman et al., 2011). There was little consistency across delineation methods, for example, DPA buffer sizes ranged from 50 m (Rodríguez et al., 2012) to over 800 m (Zenk et al., 2011), one study added an additional 20 m buffer to an MCP (Rundle et al., 2016), and one (Zenk et al., 2011; Hirsch et al., 2014; Sanchez et al., 2017) or two (Kamruzzaman and Hine, 2012) standard deviations were used for SDEs.

#### 4.1.2 Key locations (n = 13 studies)

For these studies, key locations were used to define the activity space. Locations included trip origin and destinations (van Heeswijck et al., 2015; Chaix et al., 2016, 2017; Duncan et al., 2016) destinations actively travelled to (Villanueva et al., 2012), locations for activities (Fan, 2007; Harding, 2013; Colabianchi et al., 2014; Perchoux et al., 2014; Chen and Akar, 2016; Chen, Wang and Akar, 2017; Hasanzadeh, Broberg and Kytta, 2017), and home and school addresses (Larsen et al., 2009). Measures did not capture movement between locations. MCP was commonly used to delineate activity spaces in these key location studies (n = 7). Four studies used a buffer of point locations, one of which was radial (Larsen et al., 2009) and three were network (van Heeswijck et al., 2015; Chaix et al., 2016, 2017), one study used an SDE (Perchoux et al., 2014), and one interpolated from GPS coordinates (Duncan et al., 2016).

#### 4.1.3 Specific routes or activity types (n = 12 studies)

These were typically assessed using a buffer (n = 7). Buffers of active trips or routes to and from home or school were generally smaller than those employed for other movement limits, ranging from 50 m to 500 m. One study used MCP and SDE measures to summarise the space used to make trips to or from home (Babb et al., 2017) and five studies interpolated environmental characteristics directly from point or polyline locations, describing the locations used for physical activity or passed on route (McMinn et al., 2014; Rodriguez et al., 2015; Larsen, Buliung and Faulkner, 2016; Kang et al., 2017; Quistberg et al., 2017).

### 4.2 Temporal extent of activity space

Three key elements in relation to temporality were considered when deriving activity spaces.

#### 4.2.1 Scale of data accumulation

Most activity spaces were delineated using data accumulated at the trip (n = 16), day (n = 13), or multi-day (n = 18) level. The majority of studies which assessed activity spaces at the day level used a separate measure per person per day but two studies used an average measure over a number of days to determine a mean daily activity space (Chen and Akar, 2016; Chen et al., 2017). Where specified, the minimum number of days required for participants to be included in analysis ranged from one to four. Six studies used reports of usual places visited or routes used, geo-located these and then derived activity spaces (Larsen et al., 2009; Villanueva et al., 2012; Perchoux et al., 2014; Larsen et al., 2016; Hasanzadeh et al., 2017; Yoo and Kim, 2017). Usual places were defined as those visited on a regular basis (Larsen et al., 2009; Villanueva et al., 2012; Larsen et al., 2016), were meaningful to the participant (Yoo and Kim, 2017), were visited at least once a month (Hasanzadeh et al., 2017), or had varying frequency depending on the type of destination (at least once a week, except for workplaces and supermarkets which were required to be visited for at least one third of the week or once a month respectively) (Perchoux et al., 2014).

The level of data used, whether a single trip or day, several days, or usual, appeared dependent on the research question and whether the activity space was used as an exposure or outcome. Example applications are detailed in Table 4. Often the level of aggregation was related to the temporality of other measured variables, for example, if step counts were investigated, data were often accumulated at the day level. However, authors did not always make clear the level at which data had been accumulated and justification was rarely provided. Three qualitative studies recognised that temporality may be important and investigated activity spaces for a trip, day and over several days (Vine et al., 2012; Bell et al., 2015a; Milton et al., 2015). These studies aimed to discuss specific spatial patterns of activity bouts and daily routines, how these contributed to general use of space for frequent and occasional activities over a number of days, and suggested different associations based on the temporal scale of the data.

**Table 4:** Scale of temporal data.

Level of data accumulation for deriving activity space	Example application
Trip	Estimate environmental characteristics on the route to school and investigate their associations with travel mode used on route (Helbich, 2017)
Day	Based on all activity location visited per day, estimate environmental characteristics within the activity space and their associations with the area of the activity space (Fan, 2007)
Multi-day	Based on all trips made over the course of several days, estimate walkability within activity space and investigate association with total weekly minutes of moderate physical activity (Rundle et al., 2016)
Usual	Identify ‘regular’ locations visited by individuals and estimate the size of the activity space and its association with active trips made (Perchoux et al., 2014)

Multi-day level measures aggregated movement data collected over a number of days.

#### 4.2.2 Weekday and weekend

Differences in behaviour and extent of activity for weekdays and weekends were accounted for by relatively few studies. This may be due to the limited number of days' worth of data collected, however, there is evidence of key differences in physical activity across these times (O'Donovan et al., 2017; O'Donovan et al., 2018). Only three studies quantitatively investigated the differences between weekday and weekend activity spaces (Kamruzzaman and Hine, 2012; Chen and Akar, 2016; Kang et al., 2017) with one finding that utilitarian walking was more likely to occur on weekdays than recreational walking (Kang et al., 2017). Two of the qualitative studies described how activity was patterned by day (Bell et al., 2015a; Meijering and Weitkamp, 2016) and found unique activities occurring at the weekends (Bell et al., 2015a) and differences in times and geographies of older adults' mobilities over different days depending on factors such as weather and availability of family (Meijering and Weitkamp, 2016).

Seven studies limited their investigations to weekdays as a way to capture school or work-related behaviours and two studies acknowledged the day of the week as a potential confounding factor in their analysis (Rodríguez et al., 2012; Houston, 2014), however, most studies did not account for or investigate differences in activity by day of the week.

#### 4.2.3 Exposure weighting

The extent of an individual's environmental exposure varies by the proximity to or amount of time spent in a location, and the type of activity being undertaken (Jankowska et al., 2015). For example, the same space may be experienced for longer and more closely when walking compared to when driving.

Some studies accounted for this by weighting the exposure of an environmental characteristic within an activity space. Rudimentary examples of this included limiting analysis to only locations that are frequently visited (Houston, 2014; Loebach and Gilliland, 2016a) and investigating environments experienced during a single behaviour such as walking (Rodriguez et al., 2015; Kang et al., 2017; Quistberg et al., 2017; Tribby et al., 2017). These studies assumed that the environments in which participants spent most time are the most important exposures and that environments are experienced in an equal way when undertaking the behaviour of interest. Some qualitative studies recognised clusters of activity on maps of individuals' movements and discussed reasons for ‘lingering’; identifying functional and emotional connections to regular and unique places visited (Bell et al., 2015a,b). More complex approaches used to weight exposures included cell (Helbich et al., 2016; Helbich, 2017) and inverse distance weighting (Burgoine et al., 2015) whereby a distance decay effect between individuals' recorded locations and proximate environments was applied, and a kernel density approach which weights locations by the density or duration of activity taken place there (Fan, 2007; Kamruzzaman et al., 2011; Zenk et al., 2011; van Heeswijck et al., 2015; Chaix et al., 2016, 2017).

Those studies that attempted to apply weighting techniques provided a methodological step in accounting for temporal dimensions of an individual's environmental exposure. However, the majority of studies did not consider the duration of time in any area, with some averaging measures across bouts or routes so that exposures received equal weight, irrespective of time spent in them (Rodriguez et al., 2015; Larsen et al., 2016; Kang et al., 2017). The frequency of visits to key locations was not always measured and no studies assigned different weights depending on the behaviour being undertaken. Although weighting methods were relatively uncommon and inconsistent, they highlight potential ways to capture the density of activities or identify which environmental characteristics are most strongly associated with physical activity.

### 4.3 Research questions answered

**Fig. 3:**
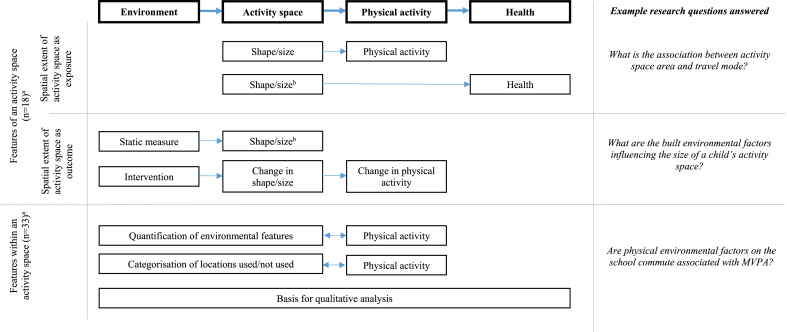
Conceptual framework of research questions answered. ^a^Totals to more than 47 as some studies address both types of research question ^b^Some studies used shape and size as an indicator of physical activity.

In describing the research questions answered by these studies, and considering our interest in eliciting how activity spaces have been used to strengthen causal inference, we categorised studies according to the research questions as they related to a possible hypothesised causal pathway (Fig. 3). This pathway might work in the following way: characteristics of the environment might influence where people are active or spend time (captured through activity spaces) which might be related to levels of physical activity and subsequent health outcomes. Studies using activity spaces addressed different research questions which mapped on to different areas of the causal pathway: studies assessed either the extent of movement by assessing the *features or parameters of the activity space itself* or used the delineation of the activity space to measure environmental *features within the activity space*.

#### 4.3.1 Features of an activity space

Eighteen studies assessed the shape and/or size of the activity space, measuring the area, perimeter or compactness as an independent or dependent variable, or moderator. Shape was measured using SDEs whilst size was derived from polygons of activity spaces. Perhaps the most straightforward application was the use of activity space parameters as an independent variable to assess the spatial extent of movement in relation to physical activity outcomes (Harding, 2013; Perchoux et al., 2014; Loebach and Gilliland, 2016; Lee et al., 2016) . Two of these studies found that a smaller and more compact activity space may be related to an increased likelihood of active travel (Harding, 2013; Perchoux et al., 2014), however, when assessing MVPA in adolescents, Lee and colleagues found that travel mode to school may be an important source of physical activity irrespective of activity space size (Lee et al., 2016). An additional study used the size of the activity space as an indicator of physical activity and reported a weak positive correlation with perceived health (Hasanzadeh et al., 2017).

Twelve studies used the activity space as an outcome to understand if access to different environmental characteristics influenced the extent of mobility and space used. Some used measures of the built environment as an independent variable and typically found that access to denser characteristics of urban form, such as walkability and connectivity, was associated with smaller activity spaces (Fan, 2007; Hirsch et al., 2014; Chen and Akar, 2016; Chen et al., 2017; Sanchez et al., 2017). Studies investigated and adjusted for sociodemographic factors and one considered the effect of weather (Fan, 2007).

Similar studies built on this relationship and investigated the role of physical activity by incorporating travel mode into their independent variables (Kamruzzaman and Hine, 2012; Loebach and Gilliland, 2016a; Vich et al., 2017) or by investigating the moderating role of public transport services (Kamruzzaman and Hine, 2012; Kamruzzaman and Hine, 2012). In the latter studies, Kamruzzaman and colleagues reported that environment-activity space relationships are sensitive to the accessibility of public transport services, car ownership and day of the week, which may be indicative of fewer travel needs or more constraints at weekends. Developing these hypotheses further, one study reported an inverse association between the presence of utilitarian destinations and activity space size but no association between activity space size and steps in school children (Villanueva et al., 2012). The lack of association observed is in contrast to other work (Harding, 2013; Perchoux et al., 2014) but aligns with findings by Lee and colleagues that suggest the size of the activity space may not be important for increasing physical activity (Lee et al., 2016). Despite the majority of these studies focusing on a narrow part of the causal pathway and differences in the strength of study design and questions answered, the general pattern of results when viewed together suggest that denser, more urban environments were associated with more contained activity spaces, and more contained activity spaces were associated with active travel.

One study assessed the area, shape, and overlap of walking-specific activity spaces with self-defined neighbourhoods before and after the development of a street design intervention designed to more safely accommodate all transport modes (Tribby et al., 2017). The authors found that walking activity spaces were significantly smaller than neighbourhoods, were included within but comprised a small proportion of the defined area, and became more compact following the intervention. Similar comparisons between activity spaces and neighbourhoods or ‘potential’ activity spaces (possible environments that could be used) are made elsewhere (Villanueva et al., 2012; Colabianchi et al., 2014; Rundle et al., 2016; Babb et al., 2017) with reports of comparable findings that walking activity spaces are smaller than neighbourhood buffers used in walkability research (Rundle et al., 2016).

#### 4.3.2 Features within an activity space

The majority of studies used activity spaces as a way to quantify environmental characteristics that populations were exposed to and then investigated the relationship between these features and physical activity (n = 33). The density or diversity of features as well as categorical descriptions of where activity had taken place (e.g inside/outside the residential neighbourhood) were used as independent variables. Measures of features were often derived from secondary and audit data, digitised in GIS, and quantified within activity space polygons or interpolated from points or routes.

The characteristics investigated varied across the studies although a number found that activity spaces with greater walkability, residential density, or utilitarian services were associated with higher MVPA and walking Larsen et al., 2009; Rodríguez et al., 2012; Duncan et al., 2016; Rundle et al., 2016; Chaix et al., 2017). However, findings were not consistent and mixed observations were reported for greenspaces and street densities (Houston, 2014; McMinn et al., 2014; van Heeswijck et al., 2015; Helbich et al., 2016; Larsen et al., 2016; Helbich, 2017). Houston and colleagues found associations between environmental features and MVPA in adults were sensitive to the proximity of features to GPS locations (Houston, 2014) which suggests observations may be dependent on the method used, as well as heterogeneity in the populations investigated. One study investigated measures of the built environment within the defined neighbourhood and walking activity space and reported that cross-sectionally, the environments within the activity space were more strongly related to walking trips but that changes to environments within the defined neighbourhood were more important for explaining changes in the number of walking trips made (Rundle et al., 2016).

Although not always made explicit in research aims, the nature of locational data used by a number of studies implied that they investigated the environments used for physical activity rather than those potentially accessible (Wheeler et al., 2010; Rodríguez et al., 2012; Houston, 2014; Kosaka et al., 2014; McMinn et al., 2014; Larsen et al., 2016; Helbich, 2017; Quistberg et al., 2017). For example, studies which characterised environments on a route described spaces that have been used for a specific behaviour or purpose. Quistberg and colleagues used the location of walking bouts to derive an activity space and estimated the risk of pedestrian collisions with motor vehicles with measures of walking (Quistberg et al., 2017). The risk of pedestrian collision is defined as an outcome and findings suggest that participants walked for recreation in areas with lower risk. However, a more interesting research question from an epidemiological perspective may be to understand how exposure to collision risk affects levels of walking and health which could plausibly be deduced from the same methodology.

Some studies addressed differences between potential access and actual usage by capturing broader spaces experienced over a day or week to identify a range of spaces that may be accessible to an individual, or by comparing features within an activity space with those accessible from a home address (Zenk et al., 2011; Chaix et al., 2016; Hirsch et al., 2016; Rundle et al., 2016). Ten studies used qualitative analysis to understand why particular environments were chosen for use (Vine et al., 2012; Bell et al., 2015b, 2015a; Milton et al., 2015; Loebach and Gilliland, 2016b; Meijering and Weitkamp, 2016; Franke et al., 2017; Plazier et al., 2017; Yoo and Kim, 2017; Hand et al., 2018).

### 4.4 Strengthening causal inference

The unit of analysis and method used to delineate an activity space gives rise to different strengths, limitations, and conceptual considerations and many studies used the most appropriate method to answer the specific research question addressed. In general, few studies discussed the implications for causal inference; however, many noted that the use of locational data beyond the residential neighbourhood was an important development in improving understanding of the causal relationships between the environment and physical activity. We used the most relevant aspects of Bradford Hill's principles of causation (Hill, 1965), to frame our synthesis of how issues were discussed and the strategies employed to deal with them. Here we focus on consistency, specificity, plausibility, temporality and experimentation.

#### 4.4.1 Consistency

The broad pattern of results suggests that dense characteristics of urban form are associated with smaller activity spaces and higher levels of physical activity. However, there is a large degree of variation in the research questions answered, methods used to derive and summarise activity spaces, environmental features identified within activity spaces and associations with activity. Some studies assessed relationships between specific behaviours and microscale features of the environment whilst others assessed more general patterns with regards to mobility. While some similarities in results were seen for studies that answered similar research questions using similar methods, as a whole there were mixed results across the entire body of literature identified.

The activity space can be used in a number of ways and applied within the same dataset to answer different but related questions. For example, Perchoux and colleagues investigated a range of questions by assessing features within the activity space and their association with the spatial dimensions of the activity space as well as assessing the features of the activity space with transport related outcomes (Perchoux et al., 2014). Findings from the different questions were consistent; showing that higher levels of active transport were associated with smaller activity spaces.

#### 4.4.2 Specificity

Delineations of the activity space typically drew on all movement or locations visited and provided little insight into how this relates to specific behaviours or whether spaces were used for physical activity. However, if the research question aims to understand how people use space, greater specificity of activity space measures might provide a stronger basis for causal inference. Daily path areas, particularly those with smaller buffer sizes, provide a more accurate estimation of space used than the SDE or MCP which can overgeneralise and lead to residual confounding (Vich et al., 2017). Although these latter measures provide a useful measure of environments potentially accessible to the individual.

#### 4.4.3 Plausibility and circularity

The use of the activity space reduces the spatial and temporal uncertainty relating to actual areas visited and time spent in locations compared to static measures of the environment, as described in the concept of the UGCP (Kwan, 2012). In most studies, the design limited the ability to understand whether spaces are used because they are supportive of a preferred activity or because they are accessible from an anchor point – the problem of selective daily mobility bias. Studies which interpolate environmental features from spatial data of a route or activity bout were at the greatest risk of selective daily mobility bias (McMinn et al., 2014; Rodriguez et al., 2015; Larsen et al., 2016; Kang et al., 2017; Quistberg et al., 2017). For example, McMinn and colleagues investigate what physical environmental characteristics are associated with MVPA on the school commute by assigning a land use category to GPS points (McMinn et al., 2014). Here, the direct environments used for travel are described, however, the environmental exposures are a direct result of individuals' travel choices which leads to an issue of circularity. Studies which use a summary measure of all locations visited provide a more plausible measure of environmental exposure, including characteristics which are both potentially accessible and used for physical activity. For example, one study characterised the percentage of parkland within a standard deviational ellipse and a buffered daily path area of all GPS trips made over the course of one week (Zenk et al., 2011). However, this measure is formulated around movement that has actually occurred and environments that individuals are exposed to as a result of their choices and does not provide any insight into where MVPA took place. Consequently, the basis for causal inference is low with respect to plausibility and circularity.

Nine studies highlighted selective daily mobility bias as an issue (Zenk et al., 2011; Kamruzzaman and Hine, 2012; Colabianchi et al., 2014; Perchoux et al., 2014; Burgoine et al., 2015; van Heeswijck et al., 2015; Chaix et al., 2016; Duncan et al., 2016; Hirsch et al., 2016) and two tried to address this by comparing potential and actual routes taken (Burgoine et al., 2015; Chaix et al., 2016). Where no significant differences were observed it was assumed that bias was minimised as route choices appeared not to be heavily based on preferences (Burgoine et al., 2015). One study controlled for selective daily mobility bias by adjusting for residential and transport preferences, as well as modes used in previous trips taken as these were thought to influence characteristics of the place visited and mode used in present trip (Chaix et al., 2016). The authors characterised trip origin and destinations but not environmental conditions along routes. This reduces the issue of circularity and by considering all destinations, provides an advance on studies which investigate environments within a residential neighbourhood. Chaix and colleagues discuss the filtering of locational data to remove locations where physical activity occurs from measures of accessible environments to mitigate bias (Chaix et al., 2013). Although this could be achieved by combining different spatial and temporal methods that are present across the studies, none of the studies in the review have attempted this.

Activity spaces were rarely used to provide evidence of plausible *mechanisms* behind observed relationships, although a number of studies used qualitative data to understand why some spaces are used and others are not. For example, Hand and colleagues used go-along interviews and personalised GPS maps to shed light on person-place transactions and commented that quantitative data could be explored further to compliment these findings (Hand et al., 2018).

#### 4.4.4 Temporality and experimentation

Studies which used aggregated momentary measures of movement, such as GPS or travel diaries, captured all locations visited over multiple days (n = 18). Conversely, some studies used self-reported measures of usual places visited explored those visited only on a regular basis (n = 6). Whilst the momentary studies capture more specific locations visited, the shorter data collection period may mean that the general pattern of behaviours are not adequately captured. Both of these are valuable depending on the research question. The distinction between these methods and the behaviour of interest should be considered in future studies and the temporal dimension of activity spaces should be well-matched to exposures or outcomes and relevant for the research question.

Little consideration was given to different temporal scales and few studies weighted exposures by length of time or type of behaviour. Consequently, it is difficult to understand whether relationships are strengthened for more proximal or longer environmental exposure.

Only four longitudinal studies were included in the review (Rodríguez et al., 2012; Franke et al., 2017; Sanchez et al., 2017; Tribby et al., 2017) which limits the causal inferences that can be made. Assessing changes in the environment, locations of activity, or anchor points over time may provide an understanding of whether this increases physical activity and could strengthen the basis for causality inference. One study used geo-referenced qualitative data to investigate why older adults chose to be active in different places (Franke et al., 2017) and another assessed temporal differences in associations between the built environment and MVPA in adolescent girls (Rodríguez et al., 2012). Both observed changes in physical activity and environmental interactions over time. However, neither considered displacement of activity due to a change in the environment.

There is an opportunity to use activity spaces in evaluative studies to complement assessments of physical activity. We identified four intervention studies which all examined environmental interventions despite our search enabling us to potentially identify individually delivered interventions. A study to assess the effect of an intervention to promote activity could use activity spaces to understand if this has changed where activity takes place or if it has changed the types of activities undertaken or with whom. It might also provide validation that changes in physical activity were directly attributable to the intervention under study. This general approach was used by Kosaka and colleagues in their assessment of covered walkways (Kosaka et al., 2014) and by Kamruzzaman and colleagues who assessed if distance to a transport service affected the size of the activity space (Kamruzzaman et al., 2011). Although both examined an intervention, study designs were cross-sectional. One study assessed changes in activity spaces and walking in response to street safety developments (Tribby et al., 2017) and it provided the strongest basis for causal inference due to its within-individual follow-up, controlled assessment of an intervention, and investigation of research questions relating to the features of and features within an activity space. Further evaluative studies of these types are required.

## 5 Recommendations for future work

Our findings illustrate that the activity space can be used to characterise the environments which people are exposed to or engage with as a result of their activities. Both the features *of* and *within* the activity space have been shown to be associated with activity but more evidence is needed to establish the direction of the causal pathway and whether the relationship between potential accessibility to environmental features and physical activity behaviours are explained by use of space. Different but complimentary research questions have been addressed and could be combined to advance the field. For example, separate methods to measure potential accessibility to environments and use of those environments could be used in the same study to answer research questions framed around understanding whether environments accessible to individuals are used for physical activity and what this means for overall activity levels.

There are a variety of spatial methods used to delineate the activity space as shown in Fig. 2, but all studies within the review captured either all movement, key locations, or locations of specific routes or activity bouts. We recommend carefully considering the distinction between measuring environments that are potentially accessible to an individual from those which the individual is directly exposed to a result of their use and using methods appropriate for the specific research question. Some studies considered differences in access and use and go some way to reducing selective daily mobility bias by comparing the activity space to residential neighbourhoods or shortest routes (Villanueva et al., 2012; Colabianchi et al., 2014; Burgoine et al., 2015; Rundle et al., 2016; Babb et al., 2017; Tribby et al., 2017). Further strategies to account for selective daily mobility bias may involve sensitivity analyses whereby separate analyses are performed for activity spaces including all behaviour and activity spaces where the behaviour or route of interest is filtered. Some authors commented on the need to understand why individuals may be active beyond their neighbourhood (Tribby et al., 2017). Future studies could improve the definition of accessibility and help unpack mechanisms to understand why some spaces are used and others are not by incorporating qualitative evidence or controlling for individuals' activity preferences.

Currently, there is little consistency in the application of temporal elements and more consideration could be given to weighting environments by their duration of use. Weights may be derived from a kernel density map of activity duration and types of activity and applied to measures of the activity space. The level of data accumulation used to derive the activity space should be appropriate for the outcome under investigation and it is important to analyse weekday and weekend relationships separately given observed differences in patterns of behaviour over these times. We identified relatively few longitudinal and intervention studies. Additional studies assessing the effects of environmental change are encouraged to strengthen casual inference and aid understanding of how interventions affect the spatial patterning of physical activity and whether levels of activity are increased, decreased, or displaced over time. We also recommend more studies in low and middle income countries to improve the generalisability of findings. This is important for understanding where physical activity occurs in different settings which could help to guide future interventions.

## 6 Strengths and limitations of the review

The strengths of this review include an extensive search strategy which was developed following an iterative process and applied to a range of specialised and interdisciplinary databases and having no restrictions on study type. The search process helped to develop and identify concepts of the activity space and the ways in which it has been used to answer questions about the relationship between environmental features, the use of those environments for activity and overall levels of physical activity. Although all included studies were in English, there was no language restriction and a number of full texts were translated to assess eligibility for inclusion. Studies published since the search may have been missed; however, the aim of the review was to describe general methods and conceptual issues which are prevalent in the literature, not to comprehensively search (Petticrew and Roberts, 2006). We discussed themes relating to study characteristics, methods and, conceptual issues in an emerging area of research that currently has little standardisation. In doing so, we highlight potential methods which could be used to answer important research questions to help researchers reduce issues of bias and strengthen causal inference, and ultimately guide future intervention research in the field.

The evidence reviewed here is complementary to evidence that describes which types of environments people are more active in, without producing a spatial summary measure. We excluded those types of studies to focus our review on the research questions addressed and methods of summarising spatial data. Some excluded studies might have strengthened the basis for causal inference in other ways. We also focused on outcomes related to activity and excluded studies using activity spaces to examine associations between environmental exposures and dietary or BMI outcomes (Burgoine et al., 2015; Cetateanu and Jones, 2016); however these studies might have important methodological contributions to add to this topic.

## 7 Conclusions

The use of the activity space is an emerging methodology for advancing studies of environment-physical activity relationships which may also be relevant for outcomes from a variety of disciplines. A range of activity space types exist and the activity space used within studies was often subject to the availability of data and the research question which the authors aimed to answer. Activity spaces can be used as both exposures and outcomes on the hypothesised environment-physical activity causal pathway and questions may relate to either the features of an activity space or features within an activity space.

There is a need for greater consistency across study designs to enable comparisons and assessment of both potentially accessible spaces and spaces used for physical activity within the same study. Longitudinal data and evaluations of interventions enabled changes in the use of space and behaviours in response to changes in the environment to be investigated, and controlling for residential and travel preferences reduced selective daily mobility bias. Currently, the application of these strategies is limited which highlights the paucity in thinking about how activity space can be used to strengthen causal inference.

## Funding

LS and JP are supported by the Medical Research Council (MC_UU_12015/6) and Centre for Diet and Activity Research (CEDAR), a UKCRC Public Health Research Centre of Excellence. Funding from the British Heart Foundation, Cancer Research UK, Economic and Social Research Council, Medical Research Council, the National Institute for Health Research (NIHR), and the Wellcome Trust, under the auspices of the UK Clinical Research Collaboration, is gratefully acknowledged (grant numbers 087636/Z/08/Z, ES/G007462/1, MR/K023187/1). LF is supported by the NIHR Global Health Research Group and Network on Diet and Activity. Funding from NIHR is gratefully acknowledged (grant number 16/137/34). No funder had any role in the study design; data collection, analysis, or interpretation; in the writing of the report; or in the decision to submit the article for publication.

## Declarations of interest

None.

## Figures and Tables

**Table A1:** Data extraction form and example study

				Population					Study type				AS measure			
Author	Year	Title	Link	Age group	Years	Sub-group	Location	Sample size	Data source	Design	Intervention	Analysis	Movement	Delineation method	Location data	Definition
Lee	2016	Does activity space size influence physical activity levels of adolescents?—A GPS study of an urban environment	Articles\ Lee_2016. pdf	Adolescent	13.8 ±0.6	N/A	Downtown Vancouver, Canada N America	39	Active Streets, Active People-Junior study, 2012	Cross-sectional	No	Quant	All trips	DPA (200 m buffer) for each person-day	GPS	Exposure: Daily AS area

Exposure			Outcome			Research Qs	Main findings	Biases and limitations of AS discussed	Additional notes
Description	Measure	Method	Description	Measure	Method				
Daily AS area	Objective	*See AS measures*	Daily MVPA (mins/day): Total, school day, trip based	Objective	Accelerometer	Is the size of activity spaces (geographic coverage of daily travel) associated with moderate-to-vigorous PA (MVPA; min/day) amongst adolescents?	There was no association between activity space size and school-day MVPA. School and school travel are important sources of PA in Vancouver adolescents, irrespective of activity space area covered.	No limitations of AS as a concept discussed	

**Table A2:** Characteristics of included studies.

Study reference		Population	Specific research question(s)	Types of environmental features assessed^b^	Measures of activity space	Assessment of physical activity	Assessment of health
Studies which investigated features of an activity space							
[5]	(Hirsch et al. (2014)	Older adults	1) Is the size/compactness of activity spaces associated with sociodemographics and available resources?	Destinations Provision for walking and cycling	Shape Size		
[11]	(Chen et al. (2017)	Adults	1) How do the effects of the built environment on activity spaces vary spatially across the study region?	Street connectivity Land use types and mix	Size		
[12]	(Lee et al. (2016)	Adolescents	1) Is the size of activity spaces associated with MVPA?		Size	MVPA	
[13]	(Fan (2007)	All	1) What effect do the built environment, traffic conditions, and weather conditions have in individual travel behaviour?	Street connectivity Natural environment	Size		
[14]	(Loebach and Gilliland (2016)	Children	1) How do individual, perceptual, or environmental factors affect the size or time children spend in neighbourhood activity space?	Street connectivity Land use types and mix	Size	Active travel	
[23]	(Harding (2013)	Adults	1) What is the relationship between activity space size and travel mode?		Size Shape	Travel mode	
[24]	(Chen and Akar (2016)	Adults	1) What are the relationships between socio-demographic characteristics, travel time, the built environment and resulting average activity spaces for all activities and non-work activities separately?	Land use types and mix	Size		
[25]	(Kamruzzaman and Hine (2012)^a^	Adults	1) Are there patterns of transport disadvantage in different rural settings identified through the activity space concept? 2) Can findings be validated with qualitative evidence from disadvantaged groups?	Street connectivity Land use types and mix	Size Shape		
[26]	(Vich et al. (2017)	Adults	1) What personal and environmental factors affect the spatial extent of daily mobility?	Street connectivity	Size	Travel mode	
[27]	(Kamruzzaman et al. (2011)	Adults	1) How does home location and socioeconomic characteristics affect the size of the activity space? 2) How does the activity space vary between those who live near a demand responsive transport route and those who do not?	Intervention Street connectivity	Size		
[40]	(Hasanzadeh et al. (2017)	Adults	1) Is size of the home range (as an indicator of physical activity) associated with perceived health?		Size		Perceived health
[42]	(Villanueva et al. (2012)	Children	1) What are the built environmental, social–cultural and individual factors influencing the size of a child's activity space?	Destinations Provision for walking and cycling Natural environment Land use types and mix	Size		
[43]	(Babb et al. (2017)^a^	Children	1) How do realised and potential activity spaces compare? 2) What features do children want/not want to use locally?		Size	Travel mode	
[45]	(Sanchez et al. (2017)	Adults	1) What are the individual, external, and contextual predictors of mobility dimensions (attributes of the activity space)?	Street connectivity Land use types and mix	Shape Size		
Studies which investigate features within an activity space							
[1]	Zenk et al. (2011)	All	1) Are features within the activity space associated with physical activity and diet?	Destinations Natural environment		MVPA	
[2]	Rodriguez et al. (2015)	Adolescents	1) Which environmental characteristics are associated with a higher likelihood of choosing a walking route? 2) How does this compare across two locations (urban/rural)?	Destinations Provision for walking and cycling Aesthetics and safety		Walking	
[3]	Rodriguez et al. (2012)	Adolescents	1) What are the associations between the outdoor built environment and MVPA? 2) How does this compare for weekends and weekdays and from one year to the next?	Destinations		LPA MVPA	
[4]	Hirsch et al. (2016)	Older adults	1) Are destinations associated with physical activity?	Destinations		Total physical activity	
[6]	Franke et al. (2017)^a^	Older adults	1) Where are older adults active and why? 2) How does this change over time?	Provision for walking and cycling		Steps	
[9]	Rundle et al. (2016)	Adults	1) What are the size and characteristics of neighbourhoods utilised and not utilised? 2) Does walkability influence the size of the utilised area? 3) Is walkability associated with physical activity in the activity space and the neighbourhood?	Provision for walking and cycling		MPA	
[10]	Burgoine et al. (2015)	Children	1) Are home, school and journey exposures to food, physical activity and built environments associated with BMI?	Destinations Provision for walking and cycling Street connectivity Aesthetics and safety Natural environment			BMI
[15]	Quistberg et al. (2017)	Adults	1) Is walking associated with pedestrian collision risk of a collision?	Street connectivity		Walking	
[16]	Houston (2014)	Adults	1) What is the relationship between built environment factors on walking routes and MVPA?	Provision for walking and cycling Natural environment Land use types and mix		Walking MVPA	
[17]	Larsen et al. (2009)	Children	1) Are characteristics of the physical and social environment associated with the journey to and from school?	Provision for walking and cycling Street connectivity Natural environment Land use types and mix		Travel mode	
[18]	Hand et al. (2018)^a^	Older adults	1) How do older adults connect within and with their neighbourhoods?	Destinations		Walking	
[19]	Larsen et al. (2016)	Children	2) Are characteristics of the built environment on the school commutes related to travel mode?	Provision for walking and cycling Street connectivity Aesthetics and safety Natural environment		Walking	
[21]	van Heeswijck et al. (2015)	Adults	1) What characteristics are associated with transport related physical activity?	Destinations Street connectivity Natural environment Land use types and mix		Active travel	
[22]	Kang et al. (2017)	Adults	1) How do recreational and utilitarian walking behaviours differ spatially and temporally?	Destinations Street connectivity Natural environment Land use types and mix		Walking	
[28]	Plazier et al. (2017)^a^	Adults	1) Under what conditions can e-bikes substitute motorised commuting? 2) Which role do travel experiences play in the daily commute by e-bike?	Provision for walking and cycling Street connectivity Aesthetics and safety		Cycling	
[29]	Chaix et al. (2017)	Adults	1) Is residential accessibility to services associated with walking? 2) Is there residential effect fallacy and confounding and can it be corrected for by using non-residential accessibility measures?	Destinations		Walking	

LPA = light physical activity, M(V)PA = moderate (to vigorous) physical activity, BMI = body mass index.

a Studies include a qualitative assessment.

b Typical features included in broad groups: **Destinations** = healthcare, community, food outlets, parks, schools, physical activity facilities, **Provision for walking and cycling** = walk score, footpath provision, cycle path provision, **Street connectivity** = connectivity, road or intersection density, public transport, **Aesthetics and safety** = aesthetics, road safety, crime safety, **Natural environment** = greenspace, bluespace, trees, slope, **Land use types and mix** = recreational, institutional, residential, commercial, urbanicity, land use mix.

## Appendix 1 Additional methodological details

### Changes from pilot searches

Pilot searches were performed in PubMed, testing different combinations of themes (GPS, physical activity, environment, and activity space), to understand if any search terms limited the results and to identify keywords from papers of interest. Titles of studies from the searches were screened and potentially relevant articles were shortlisted and reviewed with colleagues to inform the final search strategy.

After reviewing the outputs from the pilot searches and identifying articles which are suitable for achieving the aims of the review, the GPS theme was extended to capture other mapping methods and an additional theme relating to health and behaviour outcomes other than physical activity was added. The purpose of the additional subheading was to capture studies that apply the concept of the activity space, based on individuals' movement and accessible or accessed spaces, in relation to general health and socioeconomic influences of health.

Combinations of the five finalised search terms themes were tested with different Boolean operators (AND/OR) to identify which combination captured a broad range of studies, including all relevant studies identified from the pilot searches, and which returned a manageable number of results to sort through manually. In brief, using the activity space theme as an OR term returned too many records to manually sort through and using the environment theme as an OR term introduced a large number of irrelevant studies. The broad environmental terms were therefore omitted from the final search in favour of the more specific sets of terms under the mapping and activity space themes.

Google Scholar was proposed as an additional database, but the number of returned studies were unmanageable and the diversity of the topic was likely to have been captured by the general health and multidisciplinary databases: PubMed, Web of Science, TRID (Transport Research International Documentation), Scopus, Ovid MEDLINE, ProQuest and NICE (National Institute for Health and Care Excellence) evidence search.

### Pubmed search strategy

(((((((GPS OR global positioning system OR GIS OR geographical information system OR map OR mapped OR mapping OR behavioural geography OR context))

AND.

(activity space OR potential path OR daily path OR destinations)))

AND.

(((physical activity OR walk* OR bicycl* OR cycling OR exercise OR transportation OR mobility OR movement OR sport OR MVPA OR activity))

OR.

(Spatial behaviour OR health behaviour OR community OR social cohesion)))

AND Humans[Mesh]))

NOT (chromosom*[Title/Abstract] OR hippocampus[Title/Abstract] OR nervous[Title/Abstract] OR genetic*[Title/abstract] OR cortex[Title/Abstract] OR cortical[Title/Abstract] OR chemical[Title/Abstract] OR receptor[Title/abstract] OR tumor[Title/abstract] OR tumour[Title/abstract])

## APPENDIX 2 Additional Results

List of included studies

[1] S. N. Zenk et al., “Activity space environment and dietary and physical activity behaviors: a pilot study,” Health Place, vol. 17, no. 5, pp. 1150–1161, Sep. 2011.
[2] D. A. Rodriguez et al., “Influence of the Built Environment on Pedestrian Route Choices of Adolescent Girls,” Environ. Behav., vol. 47, no. 4, pp. 359–394, May 2015.
[3] D. A. Rodríguez et al., “Out and about: Association of the built environment with physical activity behaviors of adolescent females,” Health Place, vol. 18, no. 1, pp. 55–62, 2012.
[4] J. A. Hirsch, M. Winters, M. C. Ashe, P. J. Clarke, and H. A. McKay, “Destinations That Older Adults Experience Within Their GPS Activity Spaces: Relation to Objectively Measured Physical Activity,” Environ. Behav., vol. 48, no. 1, SI, pp. 55–77, Jan. 2016.
[5] J. A. Hirsch, M. Winters, P. Clarke, and H. McKay, “Generating GPS activity spaces that shed light upon the mobility habits of older adults: a descriptive analysis.,” Int. J. Health Geogr., vol. 13, p. 51, Dec. 2014.
[6] T. Franke, M. Winters, H. McKay, and H. Chaudhury, “A grounded visualization approach to explore sociospatial and temporal complexities of older adults’ mobility,” Soc. Sci. Med., vol. 193, pp. 59–69, 2017.
[7] N. Colabianchi, C. J. Coulton, J. D. Hibbert, S. M. McClure, C. E. Ievers-Landis, and E. M. Davis, “Adolescent self-defined neighborhoods and activity spaces: spatial overlap and relations to physical activity and obesity.,” Health Place, vol. 27, pp. 22–29, May 2014.
[8] C. P. Tribby, H. J. Miller, B. B. Brown, K. R. Smith, and C. M. Werner, “Geographic regions for assessing built environmental correlates with walking trips: A comparison using different metrics and model designs,” Heal. Place, vol. 45, pp. 1–9, 2017.
[9] A. G. Rundle et al., “Using GPS Data to Study Neighborhood Walkability and Physical Activity.,” Am. J. Prev. Med., vol. 50, no. 3, pp. e65–e72, Mar. 2016.
[10] T. Burgoine, A. P. Jones, R. J. Namenek Brouwer, and S. E. Benjamin Neelon, “Associations between BMI and home, school and route environmental exposures estimated using GPS and GIS: do we see evidence of selective daily mobility bias in children?,” Int. J. Health Geogr., vol. 14, p. 8, Feb. 2015.
[11] N. Chen, C.-H. Wang, and G. Akar, “Geographically Weighted Regression Approach to Investigate Spatial Variations in Activity Space,” Transp. Res. Rec. J. Transp. Res. Board, vol. 2671, pp. 40–50, Jan. 2017.
[12] N. C. Lee, C. Voss, A. D. Frazer, J. A. Hirsch, H. A. McKay, and M. Winters, “Does Activity Space Size Influence Physical Activity Levels of Adolescents? - A GPS study of an urban environment.,” Prev. Med. reports, vol. 3, pp. 75–78, 2016.
[13] Y. Fan, “The built environment, activity space, and time allocation: An activity-based framework for modeling the land use and travel connection,” The University of North Carolina at Chapel Hill, Ann Arbor, 2007.
[14] J. E. Loebach and J. A. Gilliland, “Free Range Kids? Using GPS-Derived Activity Spaces to Examine Children’s Neighborhood Activity and Mobility,” Environ. Behav., vol. 48, no. 3, pp. 421–453, Apr. 2016.
[15] D. A. Quistberg et al., “The relationship between objectively measured walking and risk of pedestrian-motor vehicle collision,” Am. J. Epidemiol., vol. 185, no. 9, pp. 810–821, 2017.
[16] D. Houston, “Implications of the modifiable areal unit problem for assessing built environment correlates of moderate and vigorous physical activity,” Appl. Geogr., vol. 50, pp. 40–47, Jun. 2014.
[17] K. Larsen, J. Gilliland, P. Hess, P. Tucker, J. Irwin, and M. He, “The Influence of the Physical Environment and Sociodemographic Characteristics on Children’s Mode of Travel to and From School.,” Am J Public Heal., vol. 99, no. 3, pp. 520–526, 2009.
[18] C. Hand, D. Rudman, S. Huot, J. Gilliland, Ma. MA, and R. Pack, “Toward Understanding Person-Place Transactions in Neighborhoods: A Qualitative-Participatory Geospatial Approach.,” Gerontologist, vol. 58, no. 1, pp. 89–100, 2018.
[19] K. Larsen, R. N. Buliung, and G. Faulkner, “School travel route measurement and built environment effects in models of children’s school travel behavior,” J. Transp. Land Use, vol. 9, no. 2, pp. 5–23, 2016.
[20] J. Loebach and J. Gilliland, “Neighbourhood play on the endangered list: examining patterns in children’s local activity and mobility using GPS monitoring and qualitative GIS,” Child. Geogr., vol. 14, no. 5, pp. 573–589, Oct. 2016.
[21] T. van Heeswijck, C. Paquet, Y. Kestens, B. Thierry, C. Morency, and M. Daniel, “Differences in associations between active transportation and built environmental exposures when expressed using different components of individual activity spaces.,” Health Place, vol. 33, pp. 195–202, May 2015.
[22] B. Kang, A. V Moudon, P. M. Hurvitz, and B. E. Saelens, “Differences in behavior, time, location, and built environment between objectively measured utilitarian and recreational walking,” Transp. Res. PART D-TRANSPORT Environ., vol. 57, pp. 185–194, Dec. 2017.
[23] C. Harding, “Activity space geometry and its effect on mode choice,” 92nd Annu. Meet. Transp. Res. Board, 2013.
[24] N. Chen and G. Akar, “Effects of neighborhood types & socio-demographics on activity space,” J. Transp. Geogr., vol. 51, pp. 112–121, 2016.
[25] M. Kamruzzaman and J. Hine, “Analysis of rural activity spaces and transport disadvantage using a multi-method approach,” Transp. POLICY, vol. 19, no. 1, pp. 105–120, Jan. 2012.
[26] G. Vich, O. Marquet, and C. Miralles-Guasch, “Suburban commuting and activity spaces: using smartphone tracking data to understand the spatial extent of travel behaviour,” Geogr. J., vol. 183, no. 4, pp. 426–439, Dec. 2017.
[27] M. Kamruzzaman, J. Hine, B. Gunay, and N. Blair, “Using GIS to visualise and evaluate student travel behaviour,” J. Transp. Geogr., vol. 19, no. 1, pp. 13–32, Jan. 2011.
[28] P. A. Plazier, G. Weitkamp, and A. E. van den Berg, “‘Cycling was never so easy!’ An analysis of e-bike commuters’ motives, travel behaviour and experiences using GPS-tracking and interviews,” J. Transp. Geogr., vol. 65, pp. 25–34, Dec. 2017.
[29] B. Chaix, D. Duncan, J. Vallee, A. Vernez-Moudon, T. Benmarhnia, and Y. Kestens, “The ‘Residential’ Effect Fallacy in Neighborhood and Health Studies Formal Definition, Empirical Identification, and Correction,” EPIDEMIOLOGY, vol. 28, no. 6, pp. 789–797, Nov. 2017.
[30] D. McMinn, N. M. Oreskovic, M. J. Aitkenhead, D. W. Johnston, S. Murtagh, and D. A. Rowe, “The physical environment and health-enhancing activity during the school commute: global positioning system, geographical information systems and accelerometry.,” Geospat. Health, vol. 8, no. 2, pp. 569–572, May 2014.
[31] M. Helbich, “Children’s school commuting in the Netherlands: Does it matter how urban form is incorporated in mode choice models?,” Int. J. Sustain. Transp., vol. 11, no. 7, pp. 507–517, 2017.
[32] M. Helbich, M. J. Z. van Emmichoven, M. J. Dijst, M.-P. Kwan, F. H. Pierik, and S. I. de Vries, “Natural and built environmental exposures on children’s active school travel: A Dutch global positioning system-based cross-sectional study,” Health Place, vol. 39, pp. 101–109, May 2016.
[33] D. T. Duncan et al., “Walk score, transportation mode choice, and walking among french adults: A GPS, accelerometer, and mobility survey study,” Int. J. Environ. Res. Public Health, vol. 13, no. 6, pp. 1–14, Jun. 2016.
[34] B. Chaix et al., “A GPS-Based Methodology to Analyze Environment-Health Associations at the Trip Level: Case-Crossover Analyses of Built Environments and Walking,” Am. J. Epidemiol., vol. 184, no. 8, pp. 579–589, Oct. 2016.
[35] S. L. Bell, C. Phoenix, R. Lovell, and B. W. Wheeler, “Using GPS and geo-narratives: a methodological approach for understanding and situating everyday green space encounters,” AREA, vol. 47, no. 1, pp. 88–96, Mar. 2015.
[36] L. Meijering and G. Weitkamp, “Numbers and narratives: Developing a mixed-methods approach to understand mobility in later life,” Soc. Sci. Med., vol. 168, no. SI, pp. 200–206, Nov. 2016.
[37] S. Milton et al., “A qualitative geographical information systems approach to explore how older people over 70 years interact with and define their neighbourhood environment.,” Health Place, vol. 36, pp. 127–133, Nov. 2015.
[38] S. L. Bell, C. Phoenix, R. Lovell, and B. W. Wheeler, “Seeking everyday wellbeing: The coast as a therapeutic landscape.,” Soc. Sci. Med., vol. 142, pp. 56–67, Oct. 2015.
[39] C. Perchoux, Y. Kestens, F. Thomas, A. Van Hulst, B. Thierry, and B. Chaix, “Assessing patterns of spatial behavior in health studies: Their socio-demographic determinants and associations with transportation modes (the RECORD Cohort Study),” Soc. Sci. Med., vol. 119, pp. 64–73, Oct. 2014.
[40] K. Hasanzadeh, A. Broberg, and M. Kytta, “Where is my neighborhood? A dynamic individual-based definition of home ranges and implementation of multiple evaluation criteria,” Appl. Geogr., vol. 84, pp. 1–10, Jul. 2017.
[41] B. W. Wheeler, A. R. Cooper, A. S. Page, and R. Jago, “Greenspace and children’s physical activity: a GPS/GIS analysis of the PEACH project.,” Prev. Med. (Baltim)., vol. 51, no. 2, pp. 148–152, Aug. 2010.
[42] K. Villanueva et al., “How far do children travel from their homes? Exploring children’s activity spaces in their neighborhood.,” Health Place, vol. 18, no. 2, pp. 263–273, Mar. 2012.
[43] C. Babb, D. Olaru, C. Curtis, and D. Robertson, “Children’s active travel, local activity spaces and wellbeing: A case study in Perth, WA,” Travel Behav. Soc., vol. 9, pp. 81–94, Oct. 2017.
[44] D. Vine, L. Buys, and R. Aird, “The use of amenities in high density neighbourhoods by older urban Australian residents,” Landsc. Urban Plan., vol. 107, no. 2, pp. 159–171, Aug. 2012.
[45] M. Sanchez et al., “Predictors of daily mobility of adults in peri-urban south India,” Int. J. Environ. Res. Public Health, vol. 14, no. 7, p. 783, Jul. 2017.
[46] S. Kosaka, M. Umezaki, M. Ishikawa, and C. Watanabe, “Physical activity and the neighborhood environment in a heavy snowfall area in Japan: The role of ‘Gangi-dori,’” Landsc. Urban Plan., vol. 123, pp. 124–133, Mar. 2014.
[47] S. Yoo and D. H. Kim, “Perceived urban neighborhood environment for physical activity of older adults in Seoul, Korea: A multimethod qualitative study,” Prev. Med. (Baltim)., vol. 103, no. S, pp. S90–S98, Oct. 2017.
